# Comparison of the analytical performance of two different electrochemical sensors based on a composite of gold nanorods with carbon nanomaterials and PEDOT:PSS for the sensitive detection of nitrite in processed meat products

**DOI:** 10.1039/d4ra04629c

**Published:** 2024-08-08

**Authors:** Wulan Tri Wahyuni, Hemas Arif Rahman, Salmi Afifah, Weni Anindya, Rayyan Azzahra Hidayat, Munawar Khalil, Bingbing Fan, Budi Riza Putra

**Affiliations:** a Department of Chemistry, Analytical Chemistry Division, Faculty of Mathematics and Natural Sciences, IPB University Bogor 16680 Indonesia; b Tropical Biopharmaca Research Center, IPB University Bogor 16680 Indonesia; c Department of Chemistry, Faculty of Mathematics and Natural Sciences, University of Indonesia Depok 16424 Indonesia; d School of Material Science and Engineering, Zhengzhou University Zhengzhou 450001 China; e Research Center for Metallurgy, National Research and Innovation Agency (BRIN) PUSPIPTEK Gd. 470 South Tangerang Banten 15315 Indonesia budi.riza.putra@brin.go.id

## Abstract

Herein, two platforms for electrochemical sensors were developed based on a combination of gold nanorods (AuNRs) with electrochemically reduced graphene oxide (ErGO) or with multiwalled carbon nanotubes (MWCNTs) and PEDOT:PSS for nitrite detection. The first and second electrodes were denoted as AuNRs/ErGO/PEDOT:PSS/GCE and AuNRs/MWCNT/PEDOT:PSS/GCE, respectively. Both materials for electrode modifiers were then characterized using UV-Vis and Raman spectroscopy, SEM, and HR-TEM. In addition, both sensors exhibit good electrochemical and electroanalytical performance for nitrite detection when investigated using voltammetric techniques. The synergistic effect between the AuNRs and their composites enhanced the electrocatalytic activity toward nitrite oxidation compared with the unmodified electrode, and the electroanalytical performance of the second electrode was superior to the first electrode. This is because the high surface area and conductivity of the MWCNTs in the second electrode provide the highest electrochemically active area (0.1510 cm^2^) among the other electrodes. Moreover, the second electrode exhibited a higher value for the surface coverage and the diffusion coefficient than the first electrode for nitrite detection. The electroanalytical performances of the first and second electrode for nitrite detection in terms of concentration range are 0.8–100 μM and 0.2–100 μM, limit of detection (0.2 μM and 0.08 μM), and measurement sensitivity (0.0451 μA μM^−1^ cm^−2^ and 0.0634 μA μM^−1^ cm^−2^). Good selectivity was also shown from both sensors in the presence of NaCl, Na_2_SO_4_, Na_3_PO_4_, MgSO_4_, NaHCO_3_, NaNO_3_, glucose, and ascorbic acid as interfering species for nitrite detection. Furthermore, both sensors were employed to detect nitrite as a food preservative in the beef sample, and the results showed no significant difference compared with the spectrophotometric technique. These results indicate that both proposed nitrite sensors may be further applied as promising electrochemical sensing platforms for *in situ* nitrite detection.

## Abstract 2

Herein, two platforms for electrochemical sensors were developed based on a combination of gold nanorods (AuNRs) with electrochemically reduced graphene oxide (ErGO) or with multiwalled carbon nanotubes (MWCNTs) and PEDOT:PSS for nitrite detection.

## Graphical abstract

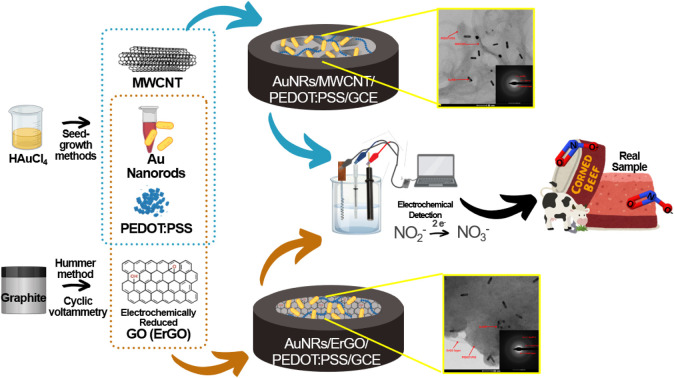

## 1 Introduction

Sodium nitrite has been widely used as a preservative in meat products due to its ability to retain the pink color related to fresh and high-quality meat to prevent lipid peroxidation at concentrations less than 150 mg kg^−1^.^1,2^ However, when sodium nitrite is applied excessively to meat products above 500 mg kg^−1^, it may be converted to carcinogenic *N*-nitrosamines and cause serious risks to human health, such as methemoglobinemia, particularly for infants and pregnant women.^3,4^ In the literature, toxic nitrite concentrations in the human body can cause methemoglobinemia at concentrations ranging from 0.4 to 200 mg per kg of body weight.^5^ Therefore, it is extremely urgent to regulate the nitrite concentration in foods, especially in meat products, to protect the health of humans worldwide. In China, the maximum nitrite concentration in dry-cured meat products is set to 30 mg kg^−1^, while in the European Union, the nitrite concentration in food products ranges from 50–250 mg kg^−1^.^6,7^ In addition, the World Health Organization (WHO) recommended an acceptable daily intake (ADI) of nitrites to the human body of 0.3 to 3.7 mg per kg of body weight.^8^ Moreover, the maximum contaminant limit (MCL) for nitrite in drinking water has been set by the United States Environmental Protection Agency (US EPA) at 1 mg L^−1^.^9^ These constraints highlight the critical need for developing a reliable and sensitive analytical method to detect nitrite in various samples to be safe for human consumption.

Numerous analytical methods have been developed for nitrite detection in several sample matrices, such as chromatography,^10,11^ spectrophotometry,^12,13^ fluorescence,^14,15^ colorimetry,^16,17^ capillary electrophoresis,^18,19^ surface-enhanced Raman scattering,^20,21^ chemiluminescence,^22,23^ and electrochemical sensors.^24,25^ Nevertheless, most of the described nitrite detection techniques have limitations, such as hazardous chemical use, sophisticated instrumentation, laborious and complex sample preparation, high cost, and limited selectivity. On the other hand, electrochemical approaches offer exciting features for development as alternative nitrite detectors due to their simplicity,^26^ rapidity,^27^ high sensitivity,^28^ low cost,^29^ and portability,^30^ which might be desirable for *in situ* analysis. However, conventional electrodes often require a large overpotential for nitrite oxidation, which eventually causes electrode fouling and poor sensitivity in complicated matrices.^31^ Consequently, some recent works have been devoted to modifying electrode surfaces by utilizing advanced conductive nanomaterials to increase the sensitivity of the current response and decrease the overpotential for nitrite oxidation. In addition, surface modification of electrodes may offer a proper way to extend the analytical range of nitrite determination during voltammetric investigations.

Various conductive nanomaterials and their composites, such as reduced graphene oxide,^32,33^ multiwalled carbon nanotubes (MWCNTs),^34,35^ metal–organic frameworks,^36,37^ metal oxides,^38,39^ MXenes,^40,41^ and layered double hydroxides,^42^ have been employed for nitrite detection to obtain a better current response than conventional electrodes. All these types of nanomaterials can be composited with conductive polymers, *e.g.*, poly(3,4-dioxythiophene):polystyrenesulfonate (PEDOT:PSS),^43,44^ polypyrrole,^45^ polythiophene,^46^ and polyaniline,^47,48^ to enhance electrode conductivity. Moreover, gold nanorods (AuNRs), as a type of gold nanoparticle, have been widely employed as modifiers in electrochemical sensing platforms due to their good biocompatibility,^49,50^ high surface area,^51,52^ and fast electron transfer ability,^53,54^ which could provide efficient mass transport properties to the electrode surface. Thus, the presence of AuNRs in composites of carbon nanomaterials and conductive polymers is expected to improve the sensitivity and selectivity of electrochemical sensors for nitrite detection.

Herein, we developed two electrochemical sensing platforms. The first platform was developed based on a composite of AuNRs, electrochemically reduced graphene oxide (ErGO), and poly(3,4-dioxythiophene):polystyrenesulfonate or PEDOT:PSS (AuNRs/ErGO/PEDOT:PSS), while the second platform was based on a composite of AuNRs, multiwalled carbon nanotubes (MWCNTs), and PEDOT:PSS (AuNRs/MWCNT/PEDOT:PSS) for nitrite detection in corned beef samples. All the material composites were then deposited on the surface of a glassy carbon electrode (GCE), and the electroanalytical performance of the resulting electrode was systematically investigated using voltammetric techniques as an electrochemical sensing platform for nitrite detection. According to the optimized conditions obtained from voltammetric studies, the AuNR/MWCNT/PEDOT:PSS/GCE exhibited higher electrocatalytic activity toward nitrite oxidation than did the AuNR/ErGO/PEDOT:PSS/GCE because of its greater surface area and conductivity. Moreover, compared with the AuNRs/ErGO/PEDOT:PSS/GCE, the AuNRs/MWCNT/PEDOT:PSS/GCE exhibits exceptional electroanalytical performance as an electrochemical sensing platform for nitrite detection with high sensitivity and a low limit of detection. The statistical analysis results also showed no significant difference in the nitrite concentration obtained from either of the proposed sensors *via* the standard UV-Vis spectrophotometric technique. Thus, it can be inferred that both proposed sensors may have the potential to be employed in practical applications for nitrite determination in food products.

## 2 Experimental

### 2.1. Reagents and apparatus

Poly(3,4-ethylenedioxythiophene):poly(styrenesulfonate) (PEDOT:PSS), H_2_SO_4_, gold(iii) chloride trihydrate (HAuCl_4_·3H_2_O), cetyltrimethylammonium bromide (CTAB), NaBH_4_, KCl, AgNO_3_, l-(+)-ascorbic acid, NaH_2_PO_4_, NaHPO_4_, glacial acetic acid, K_4_Fe(CN)_6_·3H_2_O, Zn(CH_3_COO)_2_·2H_2_O, and NaNO_2_ (CAS number: 7632-00-0) were obtained from Sigma-Aldrich. Graphite, NaNO_3_, KMnO_4_, H_2_O_2_ 30%, sulfanilamide, Na_2_B_4_O_7_·10H_2_O, and *N*-(1-naphthyl)ethylenediamine dihydrochloride (DHC) 36% were obtained from Merck, and multiwalled carbon nanotubes (MWCNTs) were obtained from Beijing Beike 2D Materials Co., Ltd, China. All the chemicals employed in this research were of analytical grade and were used without further purification. A sample of corned beef obtained from a local store was used as a real sample.

Raman spectra of graphene oxide were obtained from HORIBA HR Evolution Raman Microscopes with laser excitation at a wavelength of 514 nm. Moreover, UV-Vis-NIR spectra of gold nanorods (AuNRs) were obtained from a Thermo Scientific Genesys 10S UV-Vis Spectrophotometer (v4.007 2L5W102307). In addition, transmission electron microscopy (TEM) images of the MWCNTs and AuNRs were obtained with an FEI Tecnai G2 SuperTwin transmission electron microscope. All the electrochemical experiments were performed using a PalmSens Emstat 3 potentiostat (ES316U669) with a 3-electrode system. The three-electrode systems consisted of a glassy carbon electrode (GCE) (3 mm in diameter) from IJ Cambria Scientific as the working electrode, Ag/AgCl as the reference electrode, and Pt wire as the auxiliary electrode used in this electrochemical experiment. The standard apparatuses used in the laboratory, such as Pyrex or Iwaki glassware, a magnetic stirrer, a micropipette (Eppendorf), an analytical balance (Ohaus Instruments Co. Ltd), a pH meter (Hanna Instrument HI2210-01), a centrifuge (Hettich EBA 20), a sonicator, and an oven (Memmert GmbH), were used during the experiments.

### 2.2. Synthesis of graphene oxide

Briefly, 1 g of graphite and 0.5 g of NaNO_3_ were added to 25 mL of concentrated H_2_SO_4_ to obtain the solution mixture, which was stirred for 1 hour at 0 °C. Next, 3 g of KMnO_4_ was slowly added to the mixture, and the solution temperature was maintained below 20 °C under stirring conditions for 1 hour. Then, 50 mL of deionized water was added to the mixture to produce an exothermic reaction with a heat excess of 90–95 °C. The resulting solution was stirred for 1 hour and left for 15 minutes. Next, 50 mL of 30% H_2_O_2_ was added to the mixture and stirred for 1 hour at room temperature. The resulting solution was washed with deionized water, filtered, and dried in an oven. The obtained powder was denoted as graphene oxide (GO) and was subsequently characterized *via* Raman spectroscopy.

### 2.3. Synthesis of gold nanorods (AuNRs)

The synthesis of AuNRs was performed using the seed-growth method with some modifications.^55^ The seed solution of AuNRs was prepared by mixing 10 mL of 0.5 mM HAuCl_4_·3H_2_O with 10 mL of 0.2 M CTAB and then slowly stirring for 5 minutes. Next, 1.2 mL of 0.01 M NaBH_4_ was added to the mixture and left for 2 hours before being used for AuNR synthesis. Moreover, the growth solution was prepared by mixing 10 mL of 0.25 mM HAuCl_4_·3H_2_O with 10 mL of 0.2 M CTAB, slowly stirring for 5 minutes and allowing the mixture to stand for 10 minutes. The mixture was subsequently added to 140 μL of 0.0788 M ascorbic acid, 600 μL of 4 mM AgNO_3_, 300 μL of 1 M HCl, and 24 μL of seed solution and stirred for 20 minutes. The obtained solution was maintained at a constant temperature of 30 °C for 24 hours and then centrifuged to obtain the filtrate containing the AuNRs. The filtrate was then suspended in 0.01 M CTAB until a ruby-red color was obtained in the filtrate solution. The resulting ruby red solution is expected to contain AuNRs, which can be characterized *via* UV-Vis-NIR spectroscopy and transmission electron microscopy (TEM).

### 2.4. Preparation of the GCE modified with modified materials

Initially, each material was suspended at a concentration of 1 mg mL^−1^ in deionized water for GO and in PEDOT:PSS and isopropanol for MWCNTs. A mixture of GO/PEDOT:PSS and MWCNT/PEDOT:PSS was also prepared at a concentration of 2 mg mL^−1^, while AuNR/GO/PEDOT:PSS and AuNR/MWCNT/PEDOT:PSS were prepared by mixing GO/PEDOT:PSS or MWCNT/PEDOT:PSS 2 mg mL^−1^ with a colloidal solution of AuNRs at a ratio of 1 : 1 by volume. The GCE surface was modified with 4 μL of each material and then dried in an oven at 100 °C for 5 minutes. Next, for GO, GO/PEDOT:PSS, and AuNR/GO/PEDOT:PSS, each electrode was electrochemically reduced by applying potential scanning in the potential range from 0 to +1.3 V *vs.* Ag/Cl at a scan rate of 50 mV s^−1^ for 20 cycles. The obtained electrodes are denoted ErGO/GCE, ErGO/PEDOT:PSS/GCE, and AuNR/ErGO/PEDOT:PSS/GCE. Therefore, 10 different modified electrodes were prepared for electrochemical investigations, such as a bare GCE, GO/GCE, ErGO/GCE, MWCNT/GCE, PEDOT:PSS/GCE, GO/PEDOT:PSS/GCE, ErGO/PEDOT:PSS/GCE, MWCNT/PEDOT:PSS/GCE, AuNRs/ErGO/PEDOT:PSS/GCE, and AuNRs/MWCNT/PEDOT:PSS/GCE, for use as nitrite sensors.

### 2.5. The electrochemical behavior of the modified GCE toward nitrite sensing

The electrochemical behavior of 10 different modified electrodes (bare GCE, GO/GCE, ErGO/GCE, MWCNT/GCE, PEDOT:PSS/GCE, GO/PEDOT:PSS/GCE, ErGO/PEDOT:PSS/GCE, MWCNT/PEDOT:PSS/GCE, AuNRs/ErGO/PEDOT:PSS/GCE, and AuNRs/MWCNT/PEDOT:PSS/GCE) was evaluated by measuring 1 mM NaNO_2_ in 0.1 mM pH 7 phosphate buffer using cyclic voltammetry (CV). CV was used to investigate the electrochemical behavior of the 8 modified electrodes in a potential window from +0.4 to +1.4 V *vs.* Ag/AgCl with a scan rate of 50 mV s^−1^ in triplicate experiments. Moreover, different electrochemical techniques, such as differential pulse voltammetry (DPV), linear sweep voltammetry (LSV), and square wave voltammetry (SWV), were also applied to investigate the electrochemical performance of the materials. All voltammetric experiments were performed at a potential window from +0.1 to +1.2 V *vs.* Ag/AgCl. Moreover, DPV was performed at a scan rate of 50 mV s^−1^, a potential step (*E*_step_) of 10 mV, a pulse potential (*E*_pulse_) of 50 mV, and a pulse time (*t*_pulse_) of 0.05 s. Apart from DPV, LSV was conducted at a scan rate of 50 mV s^−1^ and an *E*_step_ of 10 mV. Furthermore, SWV was performed at a frequency of 20 Hz, an *E*_step_ of 10 mV, and an amplitude of 50 mV. From all these experiments, the peak current (*I*_pa_) and the oxidation potential (*E*_pa_) of nitrite were obtained from the modified electrodes and further used to evaluate its electrochemical performance as a nitrite sensor.

### 2.6. Evaluation of the electroanalytical performance of the selected electrode

The electroanalytical performances of the selected electrodes (AuNRs/ErGO/PEDOT:PSS/GCE and AuNRs/MWCNT/PEDOT:PSS/GCE) were evaluated for linearity, limit of detection (LOD), limit of quantitation (LOQ), sensitivity, stability, reproducibility, and selectivity. Linearity was evaluated by preparing NaNO_2_ solution in the concentration range of 10–120 μM (for AuNRs/ErGO/PEDOT:PSS/GCE) and 1–100 μM (for AuNRs/MWCNT/PEDOT:PSS/GCE) in 0.1 M pH 7 phosphate buffer. The concentration of nitrite was measured *via* DPV in triplicate at a potential window of +0.60 to +0.85 V *vs.* Ag/AgCl, a scan rate of 50 mV s^−1^, a potential step (*E*_step_) of 10 mV, a potential pulse (*E*_pulse_) of 50 mV, and a pulse time (*t*_pulse_) of 0.05 s. In addition, linearity was also investigated using chronoamperometry by applying a fixed potential (*E*_dc_) for AuNRs/ErGO/PEDOT:PSS/GCE at +0.97 V *vs.* Ag/AgCl and for AuNRs/MWCNT/PEDOT:PSS/GCE at +0.84 V *vs.* Ag/AgCl. Moreover, the LOD and LOQ were determined using the ratio of signal (S) to noise (N), specifically, the LOD (S/N ≈ 3) and LOQ (S/N ≈ 10), respectively. The sensitivity of each modified electrode was evaluated from the slope of the calibration curve for nitrite measurements. Moreover, the selectivity was investigated by adding several potential interfering species, such as NaCl, Na_2_SO_3_, Na_3_PO_4_, MgSO_4_, glucose, NaHCO_3_, and ascorbic acid, into 80 μM sodium nitrite at a concentration ratio of 1 : 1. Furthermore, the selectivity of the modified electrode was evaluated by determining the recovery value from the current response of nitrite oxidation before and after the addition of interfering species. The reproducibility of the results was studied by measuring 90 μM sodium nitrite in 0.1 M phosphate buffer at pH 7 using 5 electrodes, namely, the AuNR/ErGO/PEDOT:PSS/GCE and AuNR/MWCNT/PEDOT:PSS/GCE electrodes. In addition, the stability of the resulting material was examined by measuring 80 μM sodium nitrite in 0.1 M pH 7 phosphate buffer over 5 consecutive days *via* DPV.

### 2.7. Sample preparation

A sample of corned beef for nitrite analysis was obtained from a local store and prepared following the standard method (ISO 2918:1975).^56^ Briefly, 10 g of corned beef sample was added to 5 mL of 0.13 M Na_2_B_4_O_7_·10H_2_O and 100 mL of water at 70 °C. The mixture was heated in a water bath for 15 minutes and then cooled at room temperature. Next, 2 mL of 0.25 M K_4_Fe(CN)_6_·3H_2_O and 2 mL of Zn(CH_3_COO)_2_·2H_2_O were added to the mixture, after which the mixture was diluted in 30 mL of glacial acetic acid. The mixture was stirred thoroughly and diluted to 200 mL with deionized water. The mixture was then left for 30 minutes at room temperature. The final solution was filtered until a clear solution was obtained.

### 2.8. Nitrite detection in the corned beef sample using the proposed electrochemical method

The nitrite concentration in the obtained filtrate solution of corned beef was determined using the standard addition technique. First, 5 mL of the sample was diluted to a volume of 10 mL with the addition of NaNO_2_ at concentrations ranging from 30 to 150 μM in 0.1 M pH 7 phosphate buffer. The concentration was measured *via* DPV in triplicate at a potential window of +0.60 to +0.85 V *vs.* Ag/AgCl, a scan rate of 50 mV s^−1^, an *E*_step_ of 10 mV, an impulse of 50 mV, and a pulse of 0.05 s. The nitrite concentration in the sample of corned beef was also compared with that of the standard spectrophotometric method to determine its accuracy with that of the proposed sensor. Briefly, for the spectrophotometric method, approximately 7 mL of sample filtrate was added to deionized water until a volume of 60 mL was reached. Then, 10 mL of 0.2% w/v sulfanilamide and 6 mL of 16% v/v HCl were sequentially added to the solution, after which the mixture was thoroughly stirred. The resulting solution was allowed to stand for 5 minutes in the dark. Next, 2 mL of 0.1% w/v *N*-(1-naphthyl)ethylenediamine dihydrochloride was added to the solution, which was mixed and then left in the dark for 10 minutes. Finally, the resulting solution was diluted to a volume of 100 mL, and the absorbance was measured at a maximum wavelength of 538 nm.

## 3 Results and discussions

### 3.1. Characterization by Raman, UV-vis spectroscopy, SEM, and TEM

**Fig. 1:**
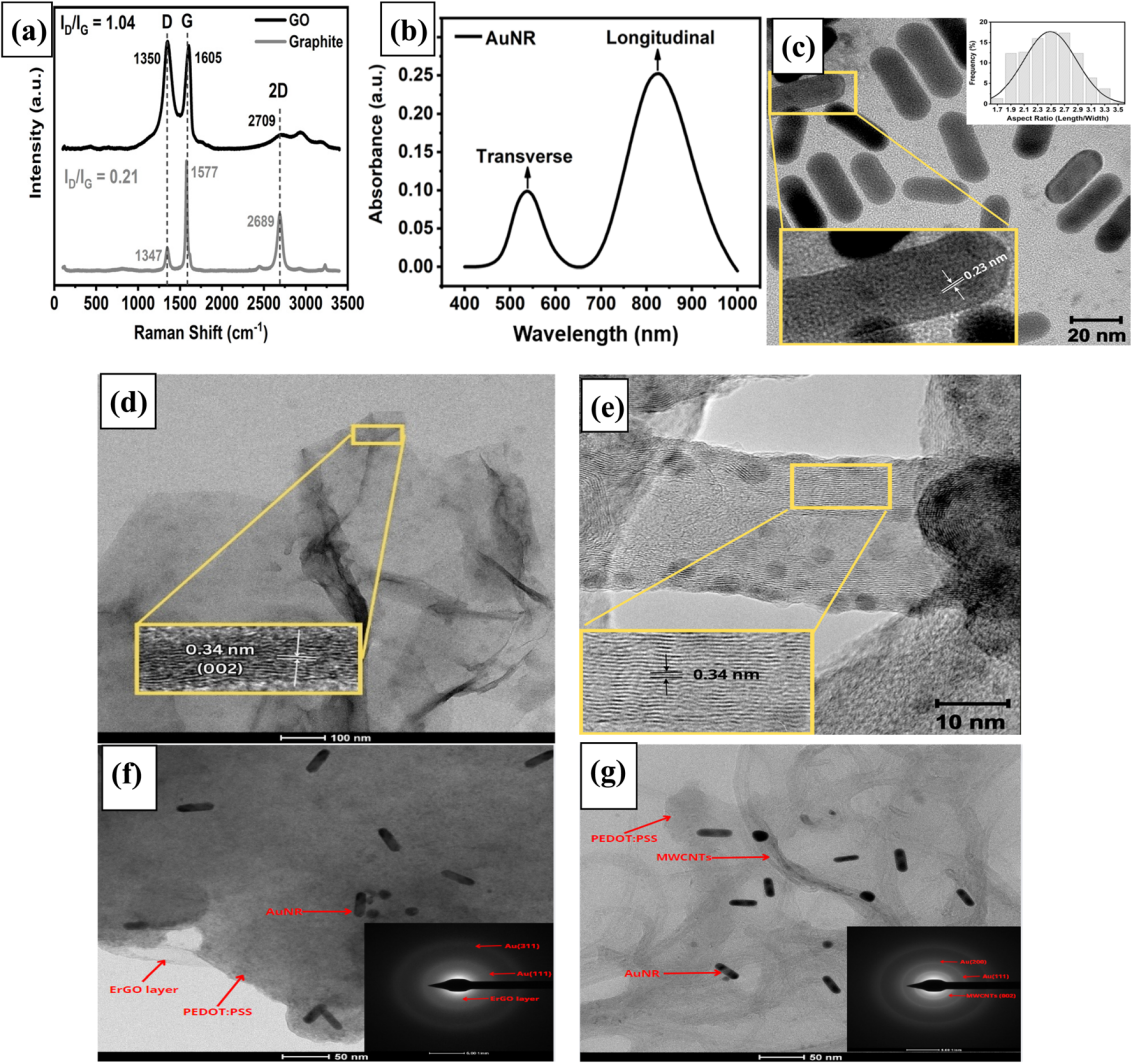
(a) Raman spectrum of graphite and synthesized graphene oxide; (b) UV-Vis spectrum of the synthesized gold nanorods (AuNRs); TEM micrograph of (c) AuNRs; inset: diameter distribution and HRTEM image of a single AuNR; (d) electrochemically reduced graphene oxide (ErGO); inset: HRTEM image; (e) HRTEM image of MWCNTs; TEM image with the SAED pattern as an inset figure for the composites; (f) AuNRs/ErGO/PEDOT:PSS; and (g) AuNRs/MWCNT/PEDOT:PSS.

The structure and number of graphene layers in graphene oxide can be investigated by Raman spectroscopy. Three typical bands are observed in the Raman spectrum, which belongs to the characteristic of carbon-based materials, *i.e.*, D, G, and 2D peaks, as shown in Fig. 1a. The D band shows the presence of defects or dislocations in the graphene layer structure. This is due to the hybridization changes of carbon atoms from sp^2^ to sp^3^ bonded with oxygen functional groups through π orbitals. Therefore, the D band at GO (1350 cm^−1^) has a higher intensity than the D band at graphite (1347 cm^−1^). Moreover, the G band corresponds to the in-plane vibrations of sp^2^-bonded carbon atoms. The position of the G band in GO (1605 cm^−1^) has shifted to a higher wavenumber with a broader peak than that in graphite (1577 cm^−1^) due to oxygenation in the GO structure.^57^ The ratio obtained from the peak intensity of D to G (*I*_D_/*I*_G_) indicates the level of defects or dislocations due to the oxidation process in the graphene layer structure. The calculated ratio of *I*_D_/*I*_G_ for graphite (0.21) improved to 1.04 when graphite was converted to GO, as shown in Fig. 1a. The third peak is the 2D band, indicating the number of graphene layers in the carbon-based materials. It is also clear from Fig. 1a that the intensity of the 2D band in GO (2709 cm^−1^) is lower than that of the 2D band in graphite (2689 cm^−1^) due to the exfoliation of graphene sheets.^58^

Gold nanoparticles have unique properties due to the resonance phenomena between light and free electrons caused by the absorption and diffraction of light at its surface.^59^ This phenomenon is known as localized surface plasmon resonance (LSPR) which is affected by the size and the morphological structure of gold nanoparticles.^60^ Due to the shape of gold nanorods (AuNRs), which are two-dimensional, two absorption bands correspond to localized surface plasmon resonance, transverse (t-LSPR) and longitudinal (l-LSPR).^61^Fig. 1b shows that the absorption band of t-LSPR is due to the oscillation of free electrons along the width of the AuNRs, which is observed in the UV-Vis region at *λ*_abs_ = 536 nm. Moreover, the l-LSPR band related to the oscillation occurs along the length of the AuNRs and is observed in the near-infrared (NIR) region at *λ*_abs_ = 830 nm. A high-intensity ratio between longitudinal and transverse LSPR bands indicates a good qualitative result of a low polydispersity in the dimensions of the gold nanorods.^62^

The morphology of the electrode modifier before being deposited onto the surface of a glassy carbon electrode (GCE) can be investigated using TEM analysis. Fig. 1c shows the morphology of the synthesized AuNRs, for which the average length and width were 32.4 ± 0.3 nm and 13.2 ± 0.1 nm, respectively. Fig. 1c also shows that the aspect ratio derived from 300 AuNR particles is between 1.7 and 3.5, with 2.5 being the highest frequency. In addition, the interplanar distance of a single AuNR can be calculated as 0.23 nm, as depicted in the inset of Fig. 1c. Moreover, the electrochemically reduced graphene oxide (ErGO) sheets (Fig. 1d) exhibit a few thin layers that are randomly aggregated, stacked with each other and have wrinkled surfaces and folded at their edges. A high-resolution TEM (HRTEM) image of ErGO revealed that the interplanar distance between the ErGO layers was 0.34 nm along the (002) plane, as displayed in the inset of Fig. 1d. Moreover, the TEM image of the MWCNTs, as depicted in Fig. 1e, shows a tubular structure with a hollow inside and a tube-like shape. The sidewall of the MWCNTs is very smooth, indicating that the graphene sheets with an outer diameter are relatively ordered and crystalline.^63^ Some of the MWCNT particles might have agglomerated due to van der Waals forces, which caused the formation of a dark layer on the tube walls, as depicted clearly in this figure. In addition, Fig. 1e shows a magnified HRTEM image of a single MWCNT with a parallel lattice fringe and a crystalline structure at its edges when examined perpendicular to the longitudinal axis of the nanotube. This could be attributed to the presence of coaxial layers in the structure of MWCNT parallel to the incident electron beam.^64^ The distance between MWCNT layers was estimated to be 0.34 nm, corresponding to the characteristics of layer separation, as shown in the inset of Fig. 1e.

Next, the morphologies of both the AuNR/ErGO/PEDOT:PSS and AuNR/MWCNT/PEDOT:PSS nanocomposites were investigated using TEM analysis and SAED patterns. Fig. 1f shows TEM image of the AuNR/ErGO/PEDOT:PSS nanocomposite, which confirms the successful integration of the PEDOT:PSS backbone into the ErGO layers occupied with AuNR particles. Further investigation of this nanocomposite using an SAED pattern (inset of Fig. 1f) also revealed that the interplanar distances for gold were 2.4 Å (111) and 1.3 Å (311) and that for ErGO was 3.6 Å (002) in the ErGO/PEDOT:PSS nanocomposite. Fig. 1g shows the TEM image of the AuNR/MWCNT/PEDOT:PSS nanocomposite, which reveals that PEDOT:PSS is coated on the structure of the MWCNTs. This coating is expected to prevent the aggregation of MWCNTs and create a homogenous solution of MWCNT/PEDOT:PSS nanocomposites.^65^ In addition, the SAED pattern of the AuNR/MWCNT/PEDOT:PSS nanocomposite shown in the inset of Fig. 1g also reveals that the interplanar distances for gold are 2.4 Å (111), 2 Å (200), and 1.3 Å (311) and that for MWCNTs is 3.4 Å (002).

**Fig. 2:**
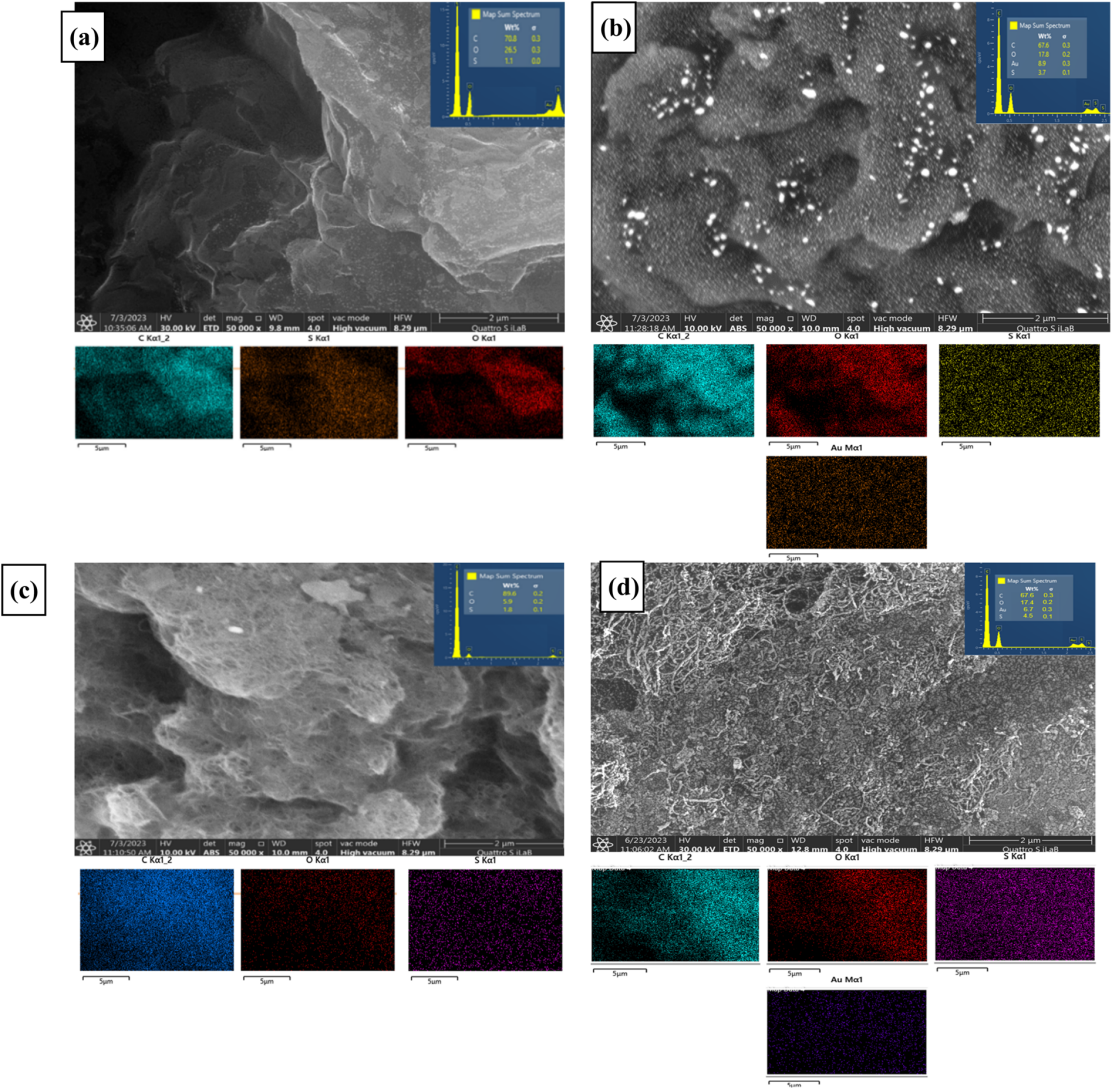
SEM images of (a) ErGO/PEDOT:PSS, (b) AuNR/ErGO/PEDOT:PSS, (c) MWCNT/PEDOT:PSS, and (d) AuNR/MWCNT/PEDOT:PSS and their related element mapping distributions of C, O, S, and Au, which were obtained from EDX analysis.

The surface morphologies of 4 different electrode modifiers (ErGO/PEDOT:PSS, AuNR/ErGO/PEDOT:PSS, MWCNT/PEDOT:PSS, and AuNR/MWCNT/PEDOT:PSS) were investigated *via* SEM analysis. Fig. 2 shows the obtained SEM images from these 4 electrode modifiers and their elemental mapping distributions. As shown in Fig. 2a and b, the ErGO/PEDOT:PSS and AuNR/ErGO/PEDOT:PSS composites exhibit a wrinkled layer structure with some cavities and sheet-like structures, which suggests a large surface area. These porous and crumpled sheet-like structures obtained from both material composites might be attributed to the formation of π–π stacking between graphene layers and the interlayer, filled by PEDOT:PSS molecules.^66^ In addition, the lower part of Fig. 2a confirms the homogenous distribution of 3 main elements on the ErGO/PEDOT:PSS surface, with a composition percentage of 70.8% C, 26.5% O, and 1.1% S. Additionally, 4 main elements (C, O, S, and Au) are well distributed on the surface of ErGO/PEDOT:PSS, as clearly depicted in the lower part of Fig. 2b. Furthermore, the percentages of these 4 elements were confirmed by EDS analysis, the values of which were 67.6% C, 17.8% O, 3.7% S, and 8.9% Au.

Fig. 2c displays the surface morphology of MWCNT/PEDOT:PSS in the presence of PEDOT:PSS as a binder to attach and be involved in the structural network with MWCNTs. In addition, it can be seen that the MWCNTs covered by PEDOT:PSS retain some nanotube cavities to increase the surface area, which is highly desirable for supporting AuNR deposition. EDX analysis was carried out to reveal a homogenous dispersion of the elemental distribution on its surface (the lower part of Fig. 2c), with a composition percentage of 89.6% C, 5.9% O, and 1.8% S. Moreover, Fig. 2d displays the occurrence of loosely coiled carbon nanotubes with a rough polymeric coating covering them, which are visible on the surface of AuNR/MWCNT-PEDOT:PSS. The presence of AuNRs in this composite is difficult to observe *via* SEM analysis due to the tendency of these materials to accumulate at microporous voids or conductive vacant regions.^67^ Furthermore, since the surface of MWCNT/PEDOT:PSS is relatively rough, AuNRs might nucleate and grow on isolated areas without merging or overlapping regions.^68^ However, the EDX analysis (the lower part of Fig. 2d) revealed a well-dispersed distribution of several constituent elements of the AuNR/MWCNT/PEDOT:PSS composite, with percentages of 67.6% C, 17.4% O, 4.5% S, and 6.7% Au.

### 3.2. Conductivity studies of 10 modified electrodes and their electrochemical behavior for nitrite detection

**Fig. 3:**
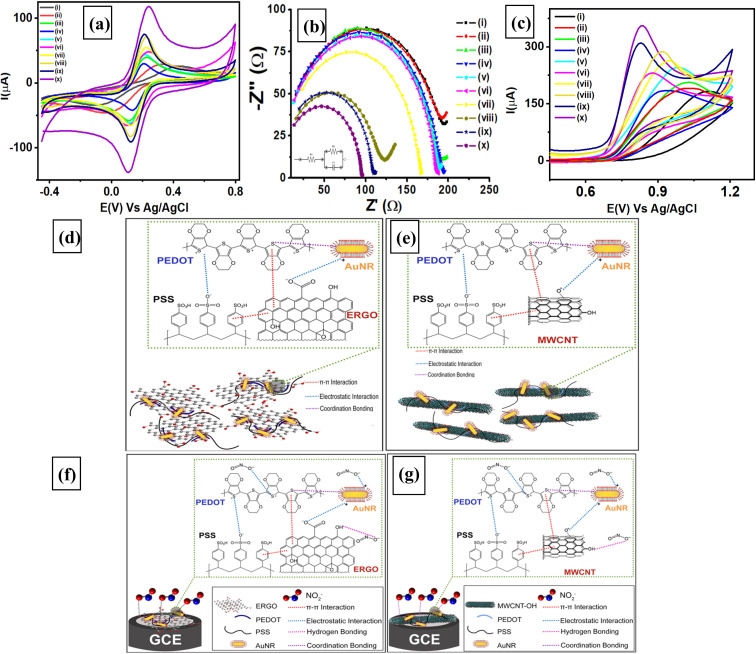
(a) Voltammogram and (b) Nyquist plot obtained from 10 different modified electrodes, *e.g.*, (i) bare GCE, (ii) GO/GCE, (iii) ErGO/GCE, (iv) PEDOT:PSS/GCE, (v) GO/PEDOT:PSS/GCE, (vi) ErGO/PEDOT:PSS/GCE, (vii) AuNRs/GO/PEDOT:PSS/GCE, (viii) MWCNT/PEDOT:PSS/GCE, (ix) AuNR/ErGO/PEDOT:PSS/GCE, and (x) AuNRs/MWCNT/PEDOT:PSS/GCE, for measuring 1 mM K_3_[Fe(CN)_6_] in a 0.1 M pH 7 phosphate buffer solution at a frequency range from 1 × 10^6^ to 5 × 10^3^ Hz, *E*_dc_ = 0 V, *E*_ac_ = 10 mV at an open circuit potential. The inset displays the equivalent circuit model for the fitting of Nyquist plot of the 10 different modified electrodes, (c) voltammograms of 5 mM NaNO_2_ in 0.1 M of pH 7 phosphate buffer obtained at a scan rate of 50 mV s^−1^ from 10 different modified electrodes, schematic illustration of the interaction between (d) ErGO/PEDOT:PSS composite with AuNRs, (e) MWCNT/PEDOT:PSS with AuNRs, interaction between NO_2_^−^ with the composite of (f) AuNR/ErGO/PEDOT:PSS (g) the composite of AuNRs/MWCNT/PEDOT:PSS for nitrite oxidation process on the surface of glassy carbon electrode (GCE).

EIS studies were performed to investigate the charge transfer resistance at the interfaces of the electrolyte/electrode interface for 10 different modified electrodes (bare GCE, GO/GCE, ErGO/GCE, PEDOT:PSS/GCE, GO/PEDOT:PSS/GCE, ErGO/PEDOT:PSS/GCE, AuNR/GO/PEDOT:PSS, AuNR/ErGO/PEDOT:PSS/GCE, MWCNT/PEDOT:PSS/GCE, and AuNR/MWCNT/PEDOT:PSS/GCE). We also displayed the voltammograms of the related Nyquist plots obtained from the 10 modified electrodes, as shown in Fig. 3a. These 10 different electrodes were analyzed by measuring 1 mM K_3_[Fe(CN)_6_] in 0.1 M pH 7 phosphate buffer with a frequency range of 1 × 10^6^ Hz to 1 × 10^3^ Hz and an *E*_ac_ = 10 mV at the open-circuit potential. The results were obtained from 10 different electrodes as semicircle regions (Nyquist plots), as indicated in Fig. 3, with different diameters of the semicircle regions. The obtained Nyquist plots for all the modified electrodes can be attributed to the resistance at the electrode/electrolyte interface and are quantitatively expressed as the value of the charge transfer resistance (*R*_2_). The *R*_2_ values of all electrodes can be obtained by fitting each Nyquist plot with an equivalent Randles circuit, as indicated in the inset of Fig. 3, which shows a downward trend in the resistance from the bare GCE to the modified electrodes. The calculated *R*_2_ values gradually decreased for the bare GCE (196 Ω), GO/GCE (193 Ω), ErGO/GCE (185 Ω), and PEDOT:PSS/GCE (181 Ω). In addition, the calculated *R*_2_ values for GO/PEDOT:PSS/GCE (178 Ω) and ErGO/PEDOT:PSS/GCE (175 Ω) indicate that these materials have a higher conductivity on the electrode surface. Interestingly, when AuNRs were deposited on the surface of the modified electrode, the electrode conductivity was enhanced, as was the case for AuNRs/GO/PEDOT:PSS (157 Ω) and AuNRs/ErGO/PEDOT:PSS (112 Ω). Ultimately, when graphene oxide was substituted with MWCNTs as an electrode modifier, the *R*_2_ values were found for MWCNT/PEDOT:PSS/GCE (105.6 Ω) and AuNR/MWCNT/PEDOT:PSS/GCE (90.49 Ω). These results indicate that the synergistic effect between AuNRs and conductive carbon materials results in electrocatalytic activity that can enhance the electron transfer process and thus significantly improve the electrochemical performance of the modified electrode. Therefore, AuNRs/ErGO/PEDOT:PSS/GCE and AuNRs/MWCNT/PEDOT:PSS/GCE were selected for further investigations as electrochemical sensing platforms for nitrite detection.

Next, the characteristics of the differently modified electrodes for electrochemical nitrite detection were investigated using cyclic voltammetry (CV). The investigation of 10 different electrodes for nitrite detection using the CV technique was performed by measuring 5 mM NaNO_2_ in 0.1 M phosphate buffer (pH 7) at a scan rate of 50 mV s^−1^, as shown in Fig. 3c. It can be seen from this figure that the electrochemical reaction of nitrite oxidation at the surface of the modified electrode results in irreversible oxidation, and the bare GCE displayed an oxidation peak potential (*E*_pa_) at 1.1 V *vs.* Ag/AgCl with a corresponding current response of 54 μA. Moreover, the current intensity of nitrite oxidation was observed at *E*_pa_ = 1.0 V *vs.* Ag/AgCl, which was obtained from GO/GCE, ErGO/GCE, and PEDOT:PSS/GCE at 35, 57, and 83 μA, respectively. In addition, 3 different electrodes, GO/PEDOT:PSS/GCE, ErGO/PEDOT:PSS/GCE, and MWCNT/PEDOT:PSS/GCE, exhibited current responses to nitrite oxidation at *E*_pa_ = 0.9 V *vs.* Ag/AgCl of 94, 126, and 149 μA, respectively. Furthermore, AuNRs/ErGO/PEDOT:PSS/GCE and AuNRs/MWCNT/PEDOT:PSS/GCE displayed more negative oxidation potentials for nitrite at 0.83 V *vs.* Ag/AgCl, with corresponding current responses of 169 and 281 μA, respectively. The shifting oxidation potential of nitrite observed at these two modified electrodes indicates a faster electron transfer process and higher conductivity among the other modified electrodes. Thus, the intensity of the anodic peak current for nitrite oxidation is 3 times greater for the AuNR/ErGO/PEDOT:PSS/GCE and 5 times greater for the AuNR/MWCNT/PEDOT:PSS/GCE than for the bare GCE. This result indicates enhanced electrocatalytic activity due to the synergistic effect between the AuNRs and the composite of carbon nanomaterials with a conductive polymer, which increased the conductivity of the modified electrodes.

### 3.3. Interaction between the electrode modifiers (AuNRs/ErGO/PEDOT:PSS and AuNRs/MWCNT/PEDOT:PSS) on the GCE surface for nitrite detection

Fig. 3d shows a schematic illustration of the ErGO/PEDOT:PSS composite due to π–π interactions between ErGO and the thiophene ring of PEDOT, which results in an enhanced conductivity of the modified electrode.^69^ This condition allows for a faster electrochemical process at the electrode surface due to the availability of channels for charge transport and thus the increasing conductivity of the ErGO/PEDOT:PSS composite.^70^ In addition, since the structure of ErGO is two-dimensional and planar, the interaction between ErGO and PEDOT:PSS may also cause the polymer chains to pack more well-arranged.^71^ Moreover, similar π–π interactions and channel effects also occur between MWCNTs and PEDOT:PSS, as displayed in Fig. 3e, as the charge density becomes more delocalized in the polymeric chains.^72^ Both interactions contributed to the improvement in the conductivity of the MWCNT/PEDOT:PSS composite at the surface of the modified electrode. However, when AuNRs were functionalized with both composites (ErGO/PEDOT:PSS and MWCNT/PEDOT:PSS), they behaved as electron channels and thus improved the electron transfer rate to the electrode surface.^73^

The interaction between AuNRs and S atoms in polythiophene chains and electrostatic interactions between gold cations and negatively charged moieties in carbon nanotubes may also occur in composites of MWCNTs/PEDOT:PSS.^74^ The AuNRs on the surface of both composites may also act as nanoscale spacers to prevent van der Waals interactions between adjacent carbon atoms in the nanomaterial structures.^75^ Consequently, a large surface area can be accessed during the electrocatalytic process at the surface of the modified electrode due to the beneficial individual properties provided by ErGO, MWCNTs, PEDOT:PSS, and AuNRs. These advantages provides a synergistic effect from each component in its material composites and could maximize the signal currents during electrochemical investigations. This leads to a higher intensity of the nitrite oxidation current, which can be attributed to the faster electron transfer process on the electrode surface compared to bare GCE. Therefore, two electrodes based on AuNRs/ErGO/PEDOT:PSS/GCE and AuNRs/MWCNT/PEDOT:PSS/GCE were selected for further use as electrochemical sensing platforms for nitrite detection.

Fig. 3f shows a schematic illustration of the chemical interactions between the electrode modifier (AuNR/ErGO/PEDOT:PSS composite) and nitrite as an analyte on the surface of the GCE. The negative charge of nitrite may be adsorbed on the surface of the modified GCE due to electrostatic interactions with positively charged PEDOT chains or with the positive charge on the surface of the AuNRs.^76^ In addition, interactions may also occur between ErGO and nitrite molecules *via* hydrogen bonding on the surface of the modified electrode. This condition facilitates nitrite accumulation at the interface of the electrode/electrolyte, which favors the electron transfer process to enhance the electrocatalytic properties of the material composite.^77^ The enhanced electrocatalytic properties of this composite may serve as a mediator to assist the electron transfer process directly to the AuNR/ErGO/PEDOT:PSS-modified electrode. Moreover, the high conductivity of MWCNTs with their excellent electron transfer capability and high surface area network, resulted in better electrical conductivity in MWCNT/PEDOT:PSS composites.^78^ The presence of AuNRs on MWCNT/PEDOT:PSS provides conduction pathways for the electron transfer processes and acts as electrocatalytic sites for nitrite oxidation on the modified electrode surface.^79^ This synergistic effect between AuNRs, MWCNTs, and PEDOT:PSS is facilitated *via* π–π interactions, electrostatic interactions, and hydrogen bonding, as shown in Fig. 3g, which contributed to the enhancement of electrocatalytic activity toward nitrite oxidation.

### 3.4. The effect of scan rate on the modified electrodes

**Fig. 4:**
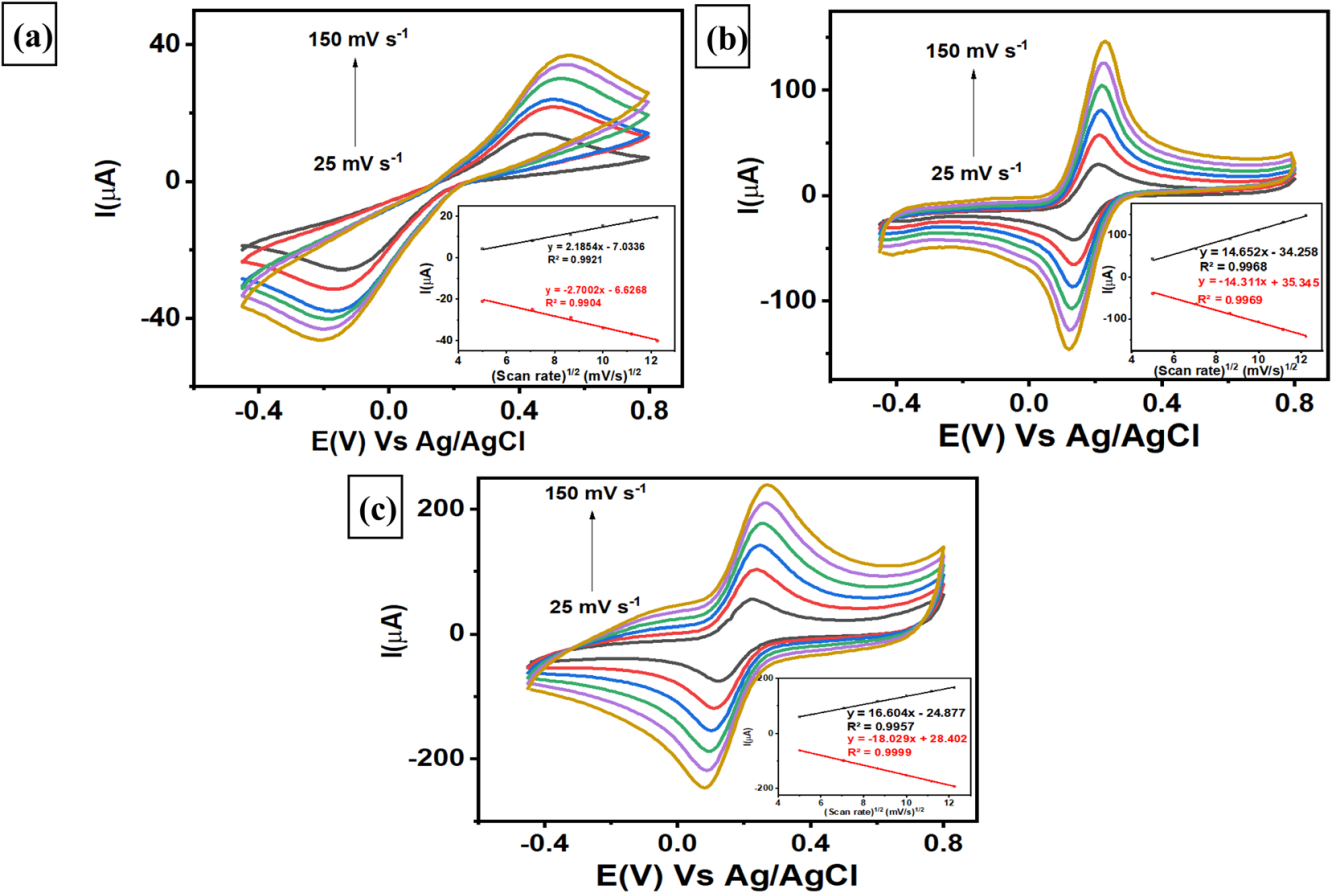
Voltammogram of 5 mM K_3_[Fe(CN)_6_] in 0.1 M KCl measured with (a) a bare GCE, (b) a AuNR/ErGO/PEDOT:PSS/GCE, and (c) a AuNR/MWCNT/PEDOT:PSS/GCE.

Investigations of the various scan rates were performed on 3 different electrodes, *i.e.*, a bare GCE, a AuNR/ErGO/PEDOT:PSS/GCE, and a AuNR/MWCNT/PEDOT:PSS/GCE, to determine the electrochemically active surface area (ECSA) of each electrode. The evaluation was conducted by measuring 5 mM K_3_[Fe(CN)_6_] in 0.1 M KCl at different scan rates from 25–150 mV s^−1^ using 3 different electrodes. As shown in Fig. 4a, the bare GCE revealed that both the anodic and cathodic peak currents increased linearly with increasing scan rate, with corresponding linear plots of *I*_pa_ (μA) = 2.1854 (mV s^−1^)^1/2^ − 7.0336, *R*^2^ = 0.9921 and *I*_pc_ (μA) = −2.7002 (mV s^−1^)^1/2^ − 6.6268, *R*^2^ = 0.9904. Moreover, the trend of the AuNRs/ErGO/PEDOT:PSS/GCE was similar to that of the corresponding linear plot, with *I*_pa_ (μA) = 14.652 (mV s^−1^)^1/2^ − 34.258, *R*^2^ = 0.9968 and *I*_pc_ (μA) = −14.311 (mV s^−1^)^1/2^ + 35.345, *R*^2^ = 0.9969, as displayed in Fig. 4b. In addition, Fig. 4c shows the corresponding calibration plot for AuNRs/MWCNT/PEDOT:PSS/GCE, in which *I*_pa_ (μA) = 16.604 (mV s^−1^)^1/2^ − 24.877, *R*^2^ = 0.9957 and *I*_pc_ (μA) = −18.029 (mV s^−1^)^1/2^ + 28.402, *R*^2^ = 0.9999. It can be inferred that all calibration plots obtained from the 3 different electrodes exhibited an excellent linear relationship between the peak current of the anodic (*I*_pa_) and cathodic (*I*_pc_) phases *versus* the square root of the scan rate. These results confirmed that the electrochemical redox reaction of [Fe(CN)_6_]^3−/4−^ species on the surface of the 3 modified electrodes was a diffusion-controlled process. Furthermore, the ECSA of each electrode can be calculated using the Randles–Sevcik eqn (1) as follows:

1 *I*_p_ = (2.69 × 10^5^)*AD*^1/2^*n*^3/2^*v*^1/2^*C*

where *I*_p_ refers to the anodic and cathodic peak current of the [Fe(CN)_6_]^3−/4−^ species (μA), *n* is the number of electrons participating in the redox reaction of [Fe(CN)_6_]^3−/4−^ species (1), *A* is the electrochemically active surface area of the electrode (cm^2^), *D* is the diffusion coefficient of the [Fe(CN)_6_]^3−/4−^ species (6.70 × 10^−6^ cm^2^ s^−1^), *v* is the scan rate (V s^−1^), and *C* is the concentration of the [Fe(CN)_6_]^3−/4−^ species (mol cm^−3^).

The ECSA of each electrode was subsequently calculated using the slope of *I*_p_*versus v*^1/2^, and the values for the bare GCE, AuNR/ErGO/PEDOT:PSS/GCE, and AuNR/MWCNT/PEDOT:PSS/GCE were 0.0138, 0.1018, and 0.1510 cm^2^, respectively. The results revealed that the ECSAs for the AuNRs/ErGO/PEDOT:PSS/GCE and AuNRs/MWCNT/PEDOT:PSS/GCE were 7.4 and 11 times greater than that of the bare GCE, respectively. Thus, it can be concluded that the ErGO/PEDOT:PSS and MWCNT/PEDOT:PSS composites could work synergistically to improve the conductivity of the modified electrode. In addition, the AuNRs on the material composite act as electron channels to accelerate the electron transfer process and act as active sites on the electrode surface. Thus, the synergistic effect between AuNRs and a material composite based on ErGO/PEDOT:PSS and MWCNT/PEDOT:PSS could improve the electrocatalytic activity of the modified electrode for use as an electrochemical sensing platform.

The respective surface coverage (*Γ*) of nitrite on the surface of AuNRs/ErGO/PEDOT:PSS/GCE and AuNRs/MWCNT/PEDOT:PSS/GCE can be calculated using the following eqn (2):2
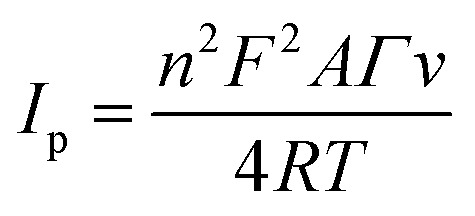
In this context, *n* signifies the number of electrons transferred, *F* represents the Faraday constant (C mol L^−1^), *A* is the electrode area (cm^2^), *Γ* is the surface coverage (mmol cm^−2^), *v* is the scan rate (V s^−1^), *R* represents the universal gas constant, and *T* pertains to the absolute temperature (K). From the slope of *I*_p_*versus v* (figure not shown), the surface coverage of nitrite adsorbed on the surface of AuNRs/ErGO/PEDOT:PSS/GCE and AuNRs/MWCNT/PEDOT:PSS/GCE were calculated to be 1.45 × 10^−6^ and 1.81 × 10^−6^ mol cm^−2^, respectively, which are comparable to those previously reported.^80,81^

### 3.5. Kinetics of the electrocatalytic activity of nitrite oxidation for both modified electrodes

**Fig. 5:**
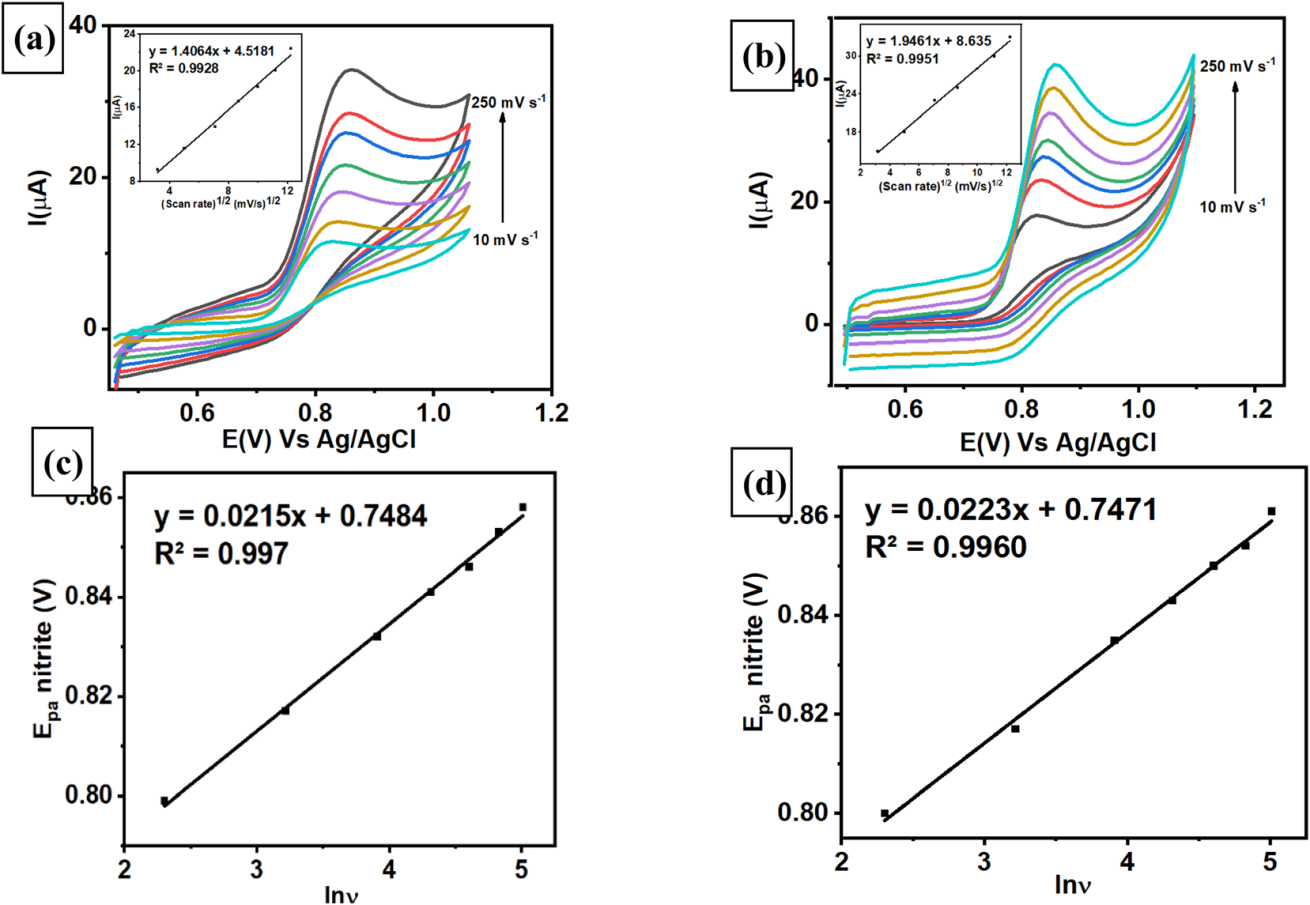
Voltammogram of 100 μM NaNO_2_ in 0.1 M pH 7 phosphate buffer obtained from (a) the AuNR/ErGO/PEDOT:PSS/GCE and (b) the AuNR/MWCNT/PEDOT:PSS/GCE. The corresponding linear plot of the logarithm of the scan rate (log *v*) *versus* the anodic peak potential (*E*_pa_) for nitrite oxidation on the (c) AuNR/ErGO/PEDOT:PSS/GCE and (d) AuNR/MWCNT/PEDOT:PSS/GCE electrodes.

The effect of scan rate was investigated by utilizing both modified electrodes to study the kinetics of electrochemical oxidation for nitrite detection using the CV technique. Fig. 5a displays the voltammograms of the AuNR/ERGO/PEDOT:PSS/GCE electrode obtained at different scan rates from 10 to 250 mV s^−1^, where the corresponding current for nitrite oxidation increased gradually. Moreover, a similar trend was observed when employing the AuNR/MWCNT/PEDOT:PSS/GCE to investigate the effect of scan rate, as displayed in Fig. 5b. The corresponding two linear relationships for both electrodes (inset of Fig. 5a and b) can be derived from eqn (3) and (4):

3 AuNRs/ErGO/PEDOT:PSS/GCE: *I*_pa_ (μA) = 1.4064 (mV s^−1^)^1/2^ + 4.5181, *R*^2^ = 0.9928

4 AuNRs/MWCNT/PEDOT:PSS/GCE: *I*_pa_ (μA) = 1.9461 (mV s^−1^)^1/2^ + 8.635, *R*^2^ = 0.9951

Thus, it can be inferred that the electrocatalytic activity on the surface of both modified electrodes is a diffusion-controlled process. Further investigations of the scan rate effect revealed the potential peak of nitrite oxidation (*E*_pa_) following the positive relationship with the natural logarithm of the scan rate (ln *v*) at different scan rates from 10 to 250 mV s^−1^. The corresponding two linear regressions derived from these studies are shown in Fig. 5c and d as follows in eqn (5) and (6):

5 AuNRs/ErGO/PEDOT:PSS/GCE: *E*_pa_ (V) = 0.0215 ln *v* (V s^−1^) + 0.7484, *R*^2^ = 0.997

6 AuNRs/MWCNT/PEDOT:PSS/GCE: *E*_pa_ (V) = 0.0223 ln *v* (V s^−1^) + 0.7471, *R*^2^ = 0.9960

The number of electrons participating in the electrochemical oxidation of nitrite on the surface of both modified electrodes can be determined using Laviron's theory as follows in eqn (7):7
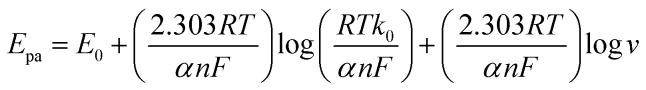
where *E*_0_ is the standard electrode potential (V), *α* is the electron transfer coefficient, *k*_0_ is the standard rate constant for the electrooxidation of nitrite (s^−1^), *T* is the absolute temperature (298 K), *R* is the universal gas constant (8.314 J K^−1^ mol^−1^), and *F* is Faraday's constant (96 495 C mol^−1^). Thus, the number of electrons involved in the electrochemical oxidation of nitrite (*n*) can be calculated from the predetermined slope of the equation (*RT*/*αnF*) with *α* = 0.5 due to irreversible reactions.^82^ Therefore, *n* was calculated to be 2.38 (≈2) for the AuNR/ErGO/PEDOT:PSS/GCE and 2.30 (≈2) for the AuNR/MWCNT/PEDOT:PSS/GCE. These results indicate that the electrocatalytic process of nitrite oxidation on the surface of both modified electrodes involves two electrons, which can be described as follows. First, nitrite loses its electron to form NO_2_ according to eqn (8), which is accompanied by a homogenous disproportionation of NO_2_ into nitrate and nitrite following eqn (9). The total reaction of nitrite conversion into nitrate can be described following the corresponding mechanism in eqn (10).

8 NO_2_^−^ → NO_2_ + e^−^

9 2NO_2_ + H_2_O → NO_3_^−^ + NO_2_^−^ + 2H^+^

10 NO_2_^−^ + H_2_O → NO_3_^−^ + 2H^+^ + 2e^−^

### 3.6. Chronoamperometric studies of the modified electrodes

**Fig. 6:**
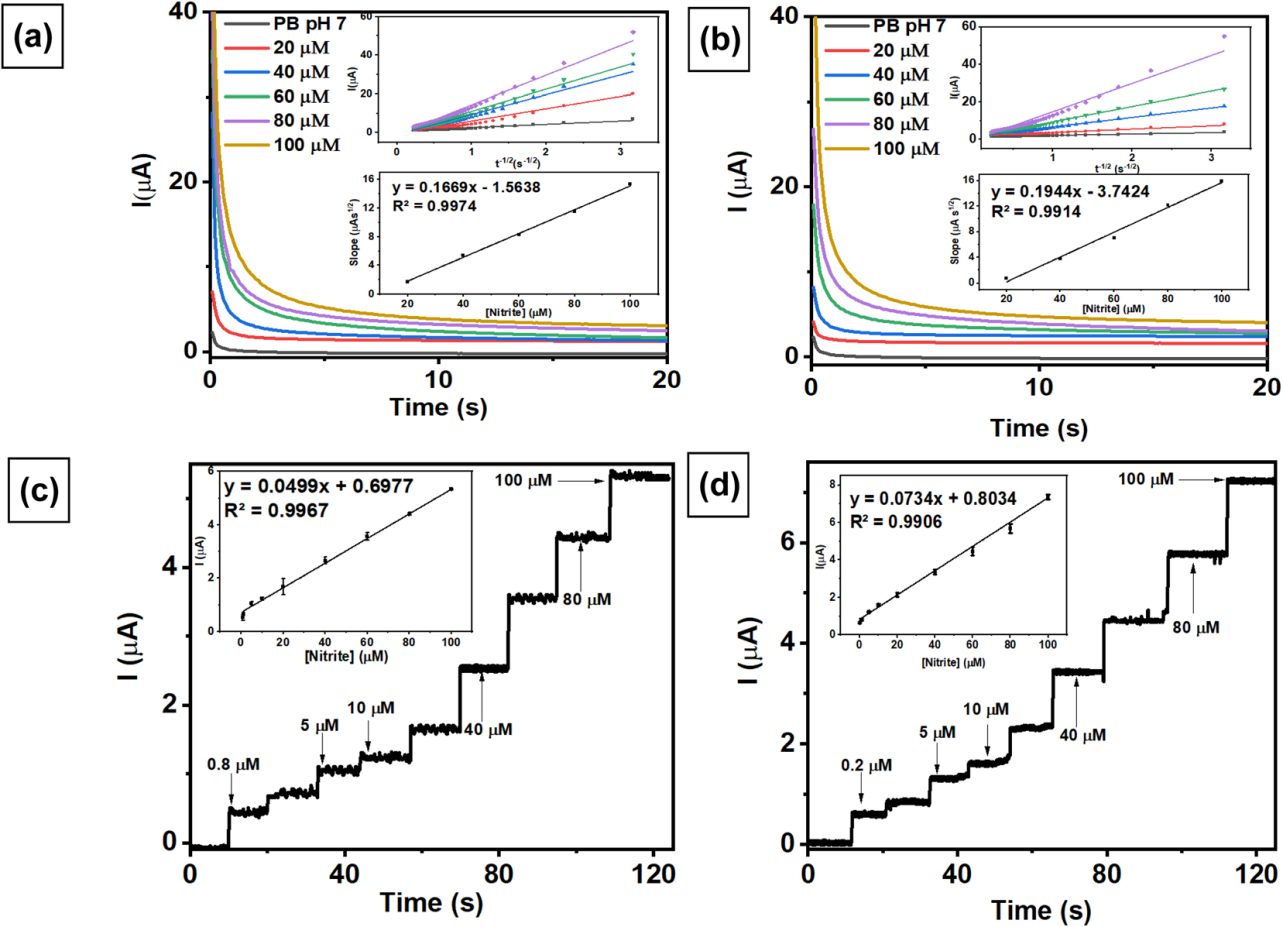
(A) Amperometric response of (a) AuNRs/ErGO/PEDOT:PSS/GCE and (b) AuNRs/MWCNT/PEDOT:PSS/GCE in 0.1 M phosphate buffer (pH 7) with different concentrations of nitrite (20–100 μM) at an applied potential of (*E*_dc_) 0.88 V *vs.* Ag/AgCl. Inset: Cottrell plot and calibration plot of the concentration of nitrite *versus* slope of the Cottrell plot. Amperograms obtained at 0.88 V *vs.* Ag/AgCl from (c) AuNRs/ErGO/PEDOT:PSS/GCE for nitrite measurements in the concentration range of 0.8–100 μM and (d) AuNRs/MWCNT/PEDOT:PSS/GCE for nitrite measurements in the concentration range of 0.2–100 μM in 0.1 M pH 7 phosphate buffer. Inset: calibration plot for the amperometric response of various nitrite concentrations *versus* their oxidation currents.

Chronoamperometric studies of the modified electrode were employed to estimate the diffusion coefficient by measuring nitrite at different concentrations. A chronoamperogram was obtained from nitrite measurements in the concentration range from 20 to 100 μM using AuNRs/ErGO/PEDOT:PSS/GCE (Fig. 6a) and AuNRs/MWCNT/PEDOT:PSS/GCE (Fig. 6b). Both chronoamperograms show an increasing anodic current of nitrite oxidation corresponding to increasing concentrations of nitrite, with the diffusion coefficient of each electrode calculated using the Cottrell equation in eqn (11).

11 *I* = *nFACD*^1/2^π^−1/2^*t*^−1/2^

where *n* is the number of electrons participating in nitrite oxidation (*n* = 2), *D* is the diffusion coefficient of nitrite (cm^2^ s^−1^), *C* is the bulk concentration of nitrite (mol cm^−3^), and *A* is the electrode area for both modified electrodes (cm^2^). From the insets of Fig. 6a and b, the plot of *I versus t*^−1/2^ for both modified electrodes was linear, and the calculated *D* values for AuNRs/ErGO/PEDOT:PSS/GCE and AuNRs/MWCNT/PEDOT:PSS/GCE were 1.66 × 10^−5^ and 2.04 × 10^−5^ cm^2^ s^−1^, respectively. These obtained values for both modified electrodes were in good agreement with previous works in the literature.^34,47^

Chronoamperometric techniques were also investigated to determine nitrite concentrations in a stirring conditions using both modified electrodes. Fig. 6c and d show the amperometric response recorded from the measurement of nitrite in 0.1 M pH 7 phosphate buffer at 0.88 V *vs.* Ag/AgCl, revealing a linear relationship related to increasing nitrite concentration. The corresponding linear relationship for the AuNR/ErGO/PEDOT:PSS/GCE electrode at nitrite concentrations ranging from 0.8–100 μM was *I*_pa_ (μA) = 0.0499 (μM) + 0.6977, *R*^2^ = 0.9967. In addition, the linear regression obtained by measuring different concentrations of nitrite from 1–100 μM using AuNRs/MWCNT/PEDOT:PSS/GCE was *I*_pa_ (μA) = 0.0734 (μM) + 0.8034, *R*^2^ = 0.9906. These results indicate that the AuNR/MWCNT/PEDOT:PSS/GCE has higher sensitivity for nitrite detection because it can operate at lower concentrations than AuNR/ErGO/PEDOT:PSS/GCE.

### 3.7. Optimization of voltammetric techniques for nitrite detection

**Fig. 7:**
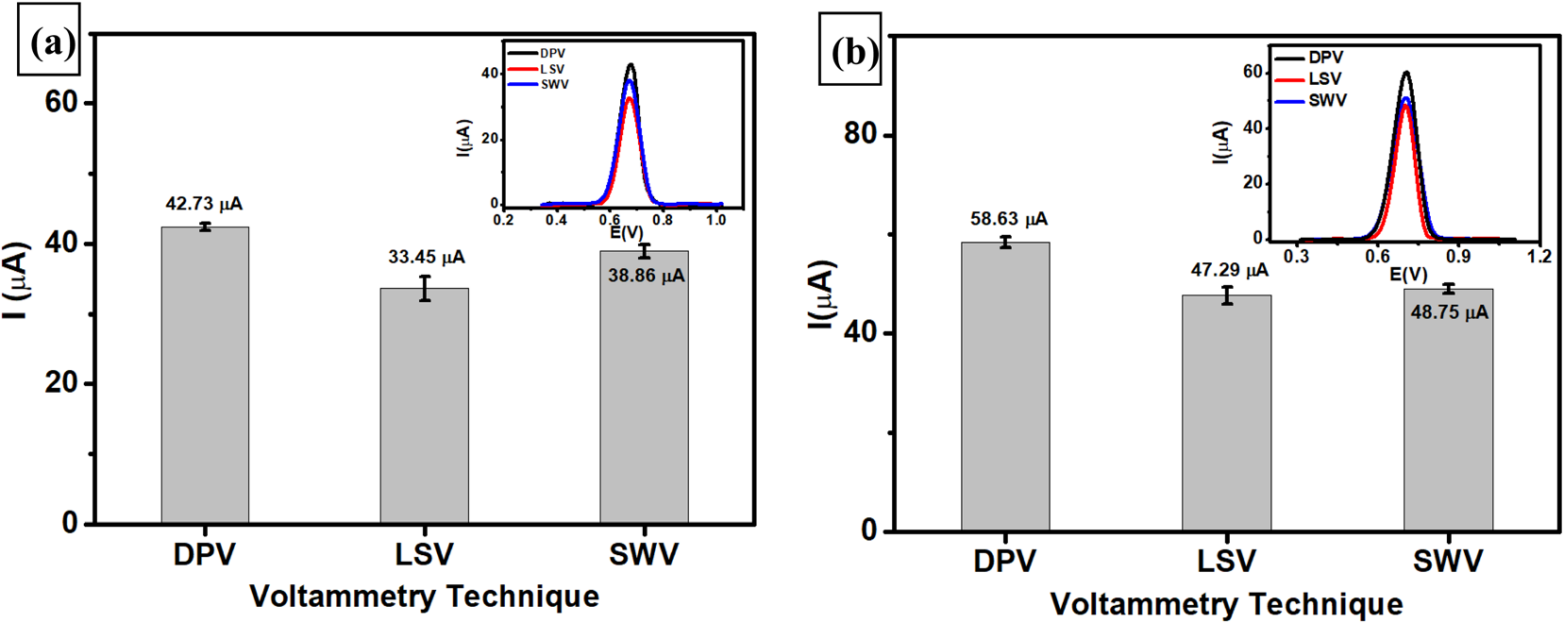
The current response measured with (a) AuNRs/ErGO/PEDOT:PSS/GCE and (b) AuNRs/MWCNT/PEDOT:PSS/GCE obtained from the measurement of 1 mM NaNO_2_ in 0.1 M of pH 7 phosphate buffer at a scan rate of 50 mV s^−1^ using 3 different voltammetric techniques: differential pulse voltammetry (DPV), linear sweep voltammetry (LSV), and square wave voltammetry (SWV).

To obtain the highest sensitivity as an electrochemical sensor, 2 modified electrodes were employed for the measurements of 1 mM NaNO_2_ in 0.1 M pH 7 phosphate buffer using 3 different voltammetric techniques, *i.e.*, differential pulse voltammetry (DPV), linear sweep voltammetry (LSV), and square wave voltammetry (SWV). Fig. 7a shows the current response of nitrite oxidation measured with AuNRs/ErGO/PEDOT:PSS/GCE using 3 different voltammetric techniques, with the highest sensitivity derived from DPV (42.73 μA) compared with SWV (38.86 μA) and LSV (33.45 μA). Moreover, the highest current was also obtained with DPV (58.63 μA) compared with that obtained with SWV (48.75 μA) and LSV (47.29 μA) when employing the AuNR/MWCNT/PEDOT:PSS/GCE for nitrite measurements, as depicted in Fig. 7b. In this case, the DPV method displays the highest sensitivity when using 2 modified electrodes, which can be attributed to the well-defined anodic peak at 0.7 V *vs.* Ag/AgCl assigned to nitrite oxidation. Therefore, the DPV technique was selected for subsequent investigations to investigate the electroanalytical performance of the modified electrodes.

### 3.8. Evaluation of the electroanalytical performance of both modified electrodes

**Fig. 8:**
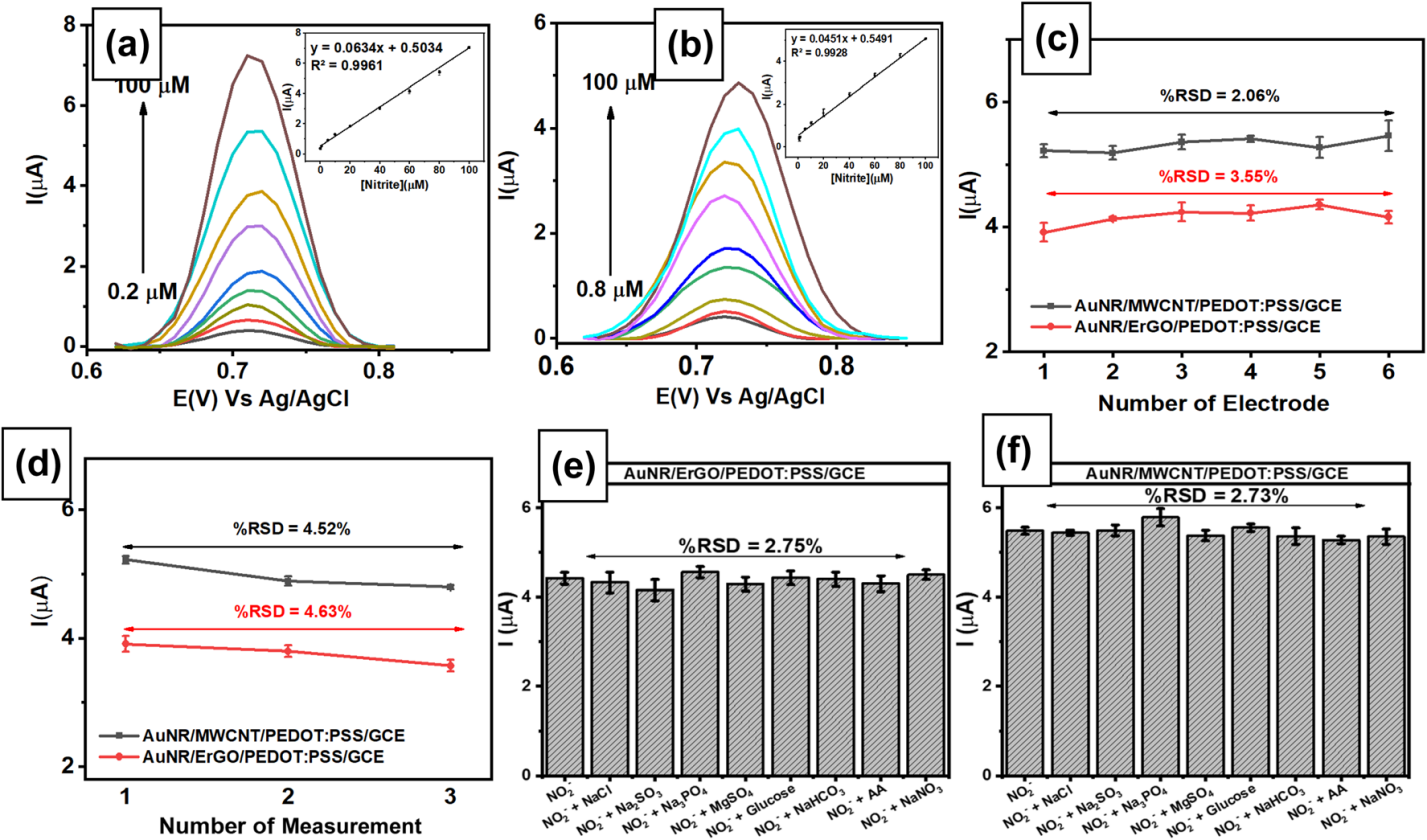
Voltammogram obtained at a scan rate of 50 mV s^−1^ from the NaNO_2_ measurements measured with (a) AuNRs/ErGO/PEDOT:PSS/GCE in the concentration range of 0.8–100 μM and (b) AuNRs/MWCNT/PEDOT:PSS/GCE in the concentration range of 0.2–100 μM. Inset: the linear relationship between the peak current and nitrite concentration; (c) Reproducibility of AuNRs/ErGO/PEDOT:PSS/GCE and AuNRs/MWCNT/PEDOT:PSS/GCE for nitrite measurements at a concentration of 80 μM using six different electrodes; (d) stability of AuNRs/ErGO/PEDOT:PSS/GCE and AuNRs/MWCNT/PEDOT:PSS/GCE for nitrite measurements at a concentration of 80 μM in three consecutive measurements; variations in the response current in the measurements of 80 μM nitrite in the presence of several potential interfering species at a concentration ratio of 1 : 1 when measured with (e) AuNRs/ErGO/PEDOT:PSS/GCE and (f) AuNRs/MWCNT/PEDOT:PSS/GCE.

To explore the electroanalytical performance of the modified electrode, differential pulse voltammetry (DPV) was used to analyze the nitrite concentration under optimum conditions. This technique was selected due to its higher sensitivity than other pulse voltammetry techniques, and it can reduce background noise when detecting low concentrations of target analytes. Nitrite measurements were performed using DPV at a scan rate of 50 mV s^−1^ in the potential range of 0.60–0.85 V in 0.1 M phosphate buffer (pH 7) as an electrolyte solution to evaluate its analytical performance, including linearity, limit of detection (LOD), and limit of quantification (LOQ). Fig. 8a displays the voltammograms at a scan rate of 50 mV s^−1^ obtained from linear studies of nitrite in the concentration range of 0.8–100 μM using the AuNR/ErGO/PEDOT:PSS/GCE. This result indicates that the peak current of nitrite oxidation proportionally increased with increasing concentration of nitrite, with the corresponding calibration plot being *I*_pa_ = 0.0451*x* + 0.5491 and *R*^2^ = 0.9928. In addition, Fig. 8b shows the results of linear studies of nitrite measurements in the concentration range from 0.2–100 μM using AuNRs/MWCNT/PEDOT:PSS/GCE with the corresponding calibration plot as *I*_pa_ = 0.0634*x* + 0.5034, *R*^2^ = 0.9961. Thus, the LOD, LOQ, and sensitivity of the AuNR/ErGO/PEDOT:PSS/GCE were 0.2 μM (1.38 × 10^−2^ μg mL^−1^), 0.8 μM (5.25 × 10^−2^ μg mL^−1^), and 0.0451 μA μM^−1^, respectively. Moreover, the LOD, LOQ, and sensitivity of the AuNR/MWCNT/PEDOT:PSS/GCE were 0.08 μM (5.25 × 10^−3^ μg mL^−1^), 0.2 μM (1.38 × 10^−2^ μg mL^−1^), and 0.0634 μA μM^−1^, respectively. Therefore, it can be concluded that the AuNR/MWCNT/PEDOT:PSS/GCE provides a higher sensitivity than does the AuNR/ErGO/PEDOT:PSS/GCE for nitrite measurements when investigated using the DPV technique. In addition, Table 1 shows a comparison of the proposed sensor based on AuNRs/ErGO/PEDOT:PSS/GCE and AuNRs/MWCNT/PEDOT:PSS/GCE with several previous works for nitrite detection. It is clear that these two modified electrodes display excellent performance, which can be attributed to the presence of AuNRs facilitating the electron transfer process combined with the conducting properties of both the ErGO/PEDOT:PSS and MWCNT/PEDOT:PSS composites on the electrode surface. These synergistic effects between metal nanoparticles and conductive materials could enhance the sensitivity of both electrochemical sensors for nitrite detection.

**Table 1:** Comparison of the performances of the proposed nitrite sensor and those of previous works

Electrode	Technique	Linear range (μM)	LOD (μM)	Sensitivity (μA μM^−1^)	Ref.
GO/PEDOT:PSS/GCE	DPV	1–200	0.5	0.027	24
MWCNTs/AuNPs/poly-melamine	DPV	10–1000	1.14	N.A	82
EdAu/SPCE^a^	SWV^b^	1–300	0.38	N.A	83
EGr/GCE^c^	SWV	0.3–1000	0.0909	9 × 10^−4^	84
Fe_3_O_4_@SiO_2_/GCE	DPV	10–1000	3.33	N.A	85
AuNF^d^/GCE	CV	0.01–5	0.01	1.966	86
Au/ZnO/ZnO@Pt-carbon cloth	CV	0.2–4986	0.09	5677	87
3D MoS_2_/2D C_3_N_4_-GCE	DPV	0.1–1100	0.065	N.A	88
Co_3_O_4_/carbon cloth	CV	1–4000	0.14	N.A	89
GNPs-SC-CPE^e^	CV	1–150	0.4	N.A	90
AuNPs/biochar/FTO	DPV	0.5–6000	0.14	1.3148	91
AuNRs/ErGO/PEDOT:PSS/GCE	DPV	0.8–100	0.2	0.0451	This work
AuNRs/MWCNT/PEDOT:PSS/GCE	DPV	0.2–100	0.08	0.0634	This work

a Electrodeposited gold on a screen-printed carbon electrode.

b Square wave voltammetry.

c Graphene/glassy carbon electrode.

d Gold nanoflower.

e Gold nanoparticles decorated on sepiolite clay.

The reproducibility of the results was evaluated by measuring 80 μM nitrite in 0.1 M phosphate buffer at pH 7 using both modified electrodes (AuNRs/ErGO/PEDOT:PSS/GCE and AuNRs/MWCNT/PEDOT:PSS/GCE). Fig. 8c shows two values of relative standard deviation (RSD) of 3.55% for AuNRs/ErGO/PEDOT:PSS/GCE and 2.06% for AuNRs/MWCNT/PEDOT:PSS/GCE, which were obtained by measuring nitrite using 6 different electrodes. Moreover, the stabilities of both modified electrodes were investigated by measuring 80 μM nitrite in 0.1 M pH 7 phosphate buffer using a similar electrode in triplicate, as shown in Fig. 8d. Based on this figure, the RSD values obtained from the AuNRs/ErGO/PEDOT:PSS/GCE and AuNRs/MWCNT/PEDOT:PSS/GCE were 4.63 and 4.52%, respectively. It can be concluded that both modified electrodes based on the AuNR/ErGO/PEDOT:PSS-modified GCE and AuNR/MWCNT/PEDOT:PSS/GCE display good reproducibility and stability for nitrite measurements and have the potential to be further studied for practical applications.

The selectivities of two proposed sensors based on AuNRs/ErGO/PEDOT:PSS/GCE and AuNRs/MWCNT/PEDOT:PSS/GCE were studied by adding several potential interfering species to address the need for selective sensing for nitrite detection. Selectivity studies were performed by adding several potential interfering species, *e.g.*, NaCl, Na_2_SO_4_, Na_3_PO_4_, MgSO_4_, NaHCO_3_, NaNO_3_, glucose, and ascorbic acid, to 80 μM nitrite at a concentration ratio of 1 : 1 using the DPV technique. As shown in Fig. 8e, the negligible effect of interfering species on the response current for nitrite oxidation measured with AuNRs/ErGO/PEDOT:PSS/GCE in triplicate experiments indicated that the %RSD was 2.72%. A similar phenomenon was also observed when AuNR/MWCNT/PEDOT:PSS/GCE (Fig. 8f) was employed for nitrite measurements in the presence of several interfering species, for which the %RSD was calculated to be 2.80% (Table 2). Thus, it can be concluded that both proposed sensors show high selectivity for use as an electrochemical sensing platform for nitrite detection.

**Table 2:** Interference effect and recovery value for determination of 80 μM nitrite in the presence of several interfering species

Electrodes	Interferences	Level of interference ratio (interference : nitrite)	*I*_nitrite_ (μA)	Recovery (%)
AuNRs/ErGO/PEDOT:PSS/GCE	—	—	4.42 ± 0.23	—
	NaCl	1 : 1	4.33 ± 0.42	98
	Na_2_SO_3_	1 : 1	4.16 ± 0.09	94
	Na_3_PO_4_	1 : 1	4.55 ± 0.22	103
	MgSO_4_	1 : 1	4.29 ± 0.27	97
	NaHCO_3_	1 : 1	4.43 ± 0.27	100
	Glucose	1 : 1	4.40 ± 0.29	99
	Ascorbic acid	1 : 1	4.30 ± 0.30	97
	Nitrate	1 : 1	4.49 ± 0.19	101
AuNRs/MWCNT/PEDOT:PSS/GCE	—	—	5.48 ± 0.14	—
	NaCl	1 : 1	5.44 ± 0.10	99
	Na_2_SO_3_	1 1	5.49 ± 0.21	100
	Na_3_PO_4_	1 : 1	5.79 ± 0.34	105
	MgSO_4_	1 : 1	5.38 ± 0.21	98
	NaHCO_3_	1 : 1	5.55 ± 0.14	101
	Glucose	1 : 1	5.37 ± 0.32	98
	Ascorbic acid	1 : 1	5.28 ± 0.14	96
	Nitrate	1 : 1	5.35 ± 0.29	97

### 3.9. Detection of nitrite in the sample of corned beef

**Fig. 9:**
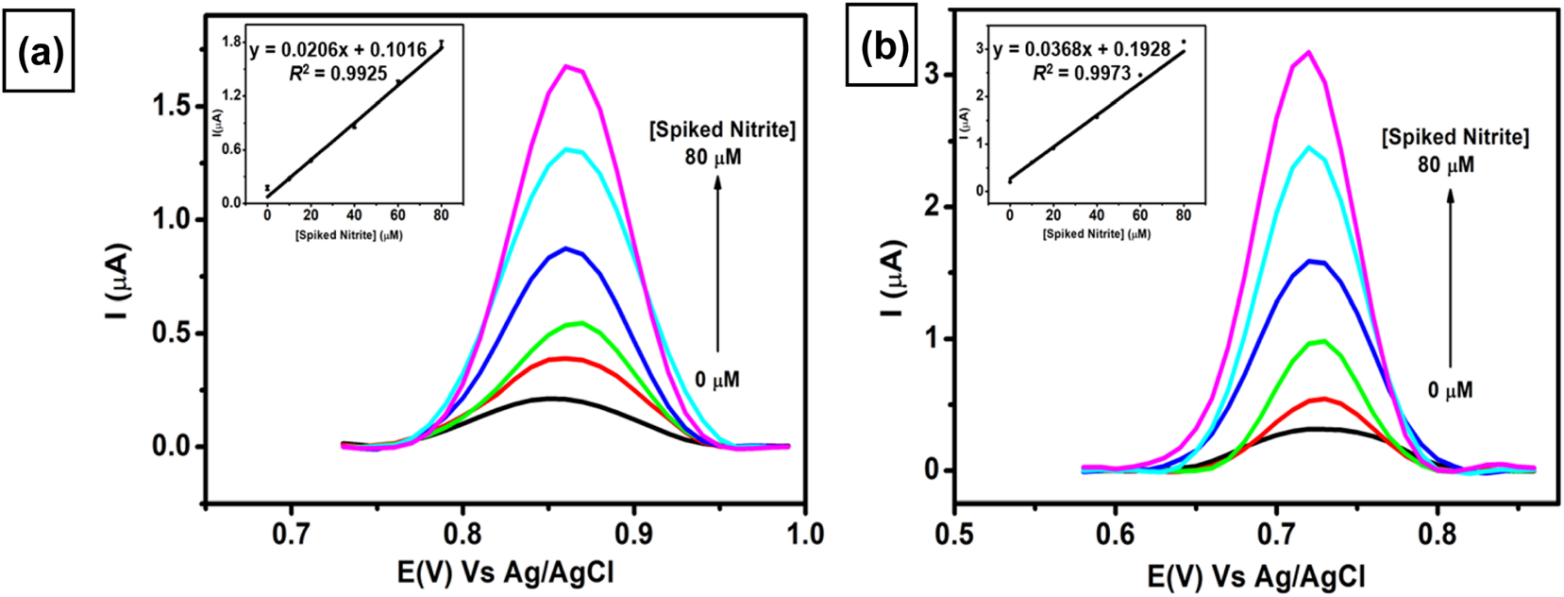
Voltammogram obtained at a scan rate of 50 mV s^−1^ for the measurement of 0.1 M pH 7 phosphate buffer containing various spiked concentrations of nitrite in the concentration range from 0–80 μM in the sample of corned beef using (a) AuNR/ErGO/PEDOT:PSS/GCE and (b) AuNR/MWCNT/PEDOT:PSS/GCE. Inset: the calibration curve derived from the multiple standard additions of nitrite to the sample of corned beef.

The applicability of two proposed sensors (AuNRs/ErGO/PEDOT:PSS/GCE and AuNRs/MWCNT/PEDOT:PSS/GCE) was evaluated by adding various concentrations of nitrite (10–80 μM) to a sample of corned beef. As displayed in Fig. 9, both proposed sensors show similar trends of increasing current response toward spiked concentration of nitrite. The two linear regressions obtained are as follows: *I*_pa_ (μA) = 0.0206 *C*_s(nitrite)_ (μA) + 0.1016, *R*^2^ = 0.9925 for AuNRs/ErGO/PEDOT:PSS/GCE (inset Fig. 9a) and *I*_pa_ (μA) = 0.0368 *C*_s(nitrite)_ (μA) + 0.1928, *R*^2^ = 0.9973 for AuNRs/MWCNT/PEDOT:PSS/GCE (inset Fig. 9b). From these two equations, the calculated nitrite concentrations in the corned beef sample obtained using AuNRs/ErGO/PEDOT:PSS/GCE were 9.853 ± 0.547 μM, and that obtained using AuNRs/MWCNT/PEDOT:PSS/GCE was 10.453 ± 0.480 μM (Table 3). In addition, the nitrite concentration in the corned beef sample was also determined *via* the standard spectrophotometric technique, and its nitrite concentration was 10.49 ± 0.383 μM. The nitrite concentrations obtained with both electrochemical and spectrophotometric methods were carried out in triplicate experiments to evaluate their precision. The concentrations obtained from both of these techniques were statistically compared using Student's *t*-test at the 95% confidence interval, and there was no significant difference between the two methods. In addition, Table 3 also shows that the nitrite concentrations obtained from these two methods are lower than the maximum concentration recommended by the Codex Committee on Food Additives in 2021.^92^ Therefore, it can be inferred that both proposed sensors could be employed for nitrite determination in samples of meat products, and their results are comparable with those of the standard method. These results also confirmed that the nitrite content in the sample of processed meat is within the safe limit for human consumption.

**Table 3:** Comparison of nitrite concentrations in corned beef samples determined with electrochemical and spectrophotometric techniques

Method	Concentration (μM)	Maximum concentration (μM) (CCFA 2021)
AuNRs/ErGO/PEDOT:PSS/GCE	9.853 ± 0.547	21.74
AuNRs/MWCNT/PEDOT:PSS/GCE	10.453 ± 0.480	21.74
Spectrophotometric	10.49 ± 0.383	21.74

## 4 Conclusions

In summary, we have successfully demonstrated the fabrication of two electrochemical sensors based on two different composites (AuNRs/ErGO/PEDOT:PSS and AuNRs/MWCNT/PEDOT:PSS) for nitrite detection. Gold nanorods were synthesized using the seed-growth method and then characterized using UV-Vis spectroscopy and TEM to obtain an average aspect ratio of 2.5. Both composites based on AuNRs/ErGO/PEDOT:PSS and AuNRs/MWCNT/PEDOT:PSS were further characterized using SEM and TEM to investigate their surface morphologies. In addition, EIS studies were employed to study the electrode resistance and revealed that two proposed sensors based on AuNRs/ErGO/PEDOT:PSS/GCE and AuNRs/MWCNT/PEDOT:PSS/GCE displayed the highest conductivity among the other electrodes. Furthermore, when both proposed sensors were employed for nitrite detection, the current intensities for the AuNRs/ErGO/PEDOT:PSS/GCE and AuNRs/MWCNT/PEDOT:PSS/GCE were 3 and 5 times greater than that of the bare GCE, respectively. The electroanalytical performance of the AuNRs/ErGO/PEDOT:PSS/GCE was linear to nitrite concentration (0.8–100 μM), a low limit of detection (1.38 × 10^−2^ μg mL^−1^) and high sensitivity (0.0451 μA μM^−1^). Moreover, the AuNRs/MWCNT/PEDOT:PSS/GCE displayed better electroanalytical performance over a wider range of nitrite concentrations (0.2–100 μM), with a lower limit of detection (5.25 × 10^−3^ μg mL^−1^) and higher sensitivity (0.0634 μA μM^−1^) than did the AuNRs/ErGO/PEDOT:PSS/GCE. The enhanced conductivity of both modified electrodes is due to the synergistic effect between gold nanorods acting as “electronic wires” to provide channels for the electron transfer process and either ErGO/PEDOT:PSS or MWCNT/PEDOT:PSS acting as conductive nanomaterials on the GCE surface. Both modified electrodes displayed excellent stability and selectivity for nitrite measurements in the presence of several interfering species, such as ionic compounds and organic molecules. For practical application, both proposed sensors were also employed for nitrite measurements in the sample of processed meat (corned beef), and the results showed no significant difference compared with those of the standard spectrophotometric technique according to Student's *t*-test at the 95% confidence interval. Therefore, both proposed sensors provide alternative methods for nitrite determination and can be further explored for other types of preservatives in food products.

## Data availability

The authors confirm that the data supporting the findings of this study are available within the article.

## Author contributions

Writing, supervision, review, funding acquisition: Wulan Tri Wahyuni. Data acquisition: Hemas Arif Rahman, Salmi Afifah, Weni Anindya, Rayyan Azzahra Hidayat. Conceptualization, supervision: Munawar Khalil. Conceptualization: Bingbing Fan. Writing – original draft, supervision, review, conceptualization: Budi Riza Putra.

## Conflicts of interest

There are no conflicts to declare.
